# Gastrointestinal parasite infections in fighting bulls in South Thailand

**DOI:** 10.14202/vetworld.2020.1544-1548

**Published:** 2020-08-10

**Authors:** Domechai Kaewnoi, Ratchakul Wiriyaprom, Saowakon Indoung, Ruttayaporn Ngasaman

**Affiliations:** Faculty of Veterinary Science, Prince of Songkla University, Hatyai, Songkhla, Thailand

**Keywords:** fighting bulls, gastrointestinal parasite, southern, Thailand

## Abstract

**Background and Aim:**

Bullfighting is booming in South Thailand, attracting tourists, and stimulating local economies. The bulls are well raised and practiced, but in many cases, the owners lack knowledge and understanding of the prevention of animal diseases, including parasitic infections. This study aimed to determine the occurrence of gastrointestinal (GI) parasite infection in fighting bulls.

**Materials and Methods:**

A total of 1501 fecal samples were collected from bulls aged 2-5 years visiting the animal hospital of Prince of Songkla University during 2016-2019. The formalin ethylether concentration method was used to detect GI parasites in feces.

**Results:**

The overall rate of GI parasite infection was 94.27%. Rumen fluke eggs were detected in 97.17% of all infected animals, followed in prevalence by strongyles (26.29%), *Eurytrema* spp. (2.83%), *Fasciola* spp. (2.47%), *Trichuris* spp. (0.35%), and *Moniezia* spp. (0.14%). Two protozoan genera were identified, *Balantidium coli* (6.64%) and *Eimeria* spp. (3.53%). Coinfection was observed in 33.99%. The five most common coinfections were rumen fluke with strongyles (20.85%), *B. coli* (4.66%), *Eimeria* spp. (1.55%), *Eurytrema* spp. (1.34%), and *Fasciola* spp. (1.06%).

**Conclusion:**

In addition to high GI parasite infection rates, zoonotic parasites were observed. Therefore, it is recommended that farmers should follow good sanitation and prevention practices to control parasitic infections in bulls, and proper hygienic precautions should be taken by the owners. Implementation of deworming programs using appropriateanthelmintic drugs as well as rotation of anthelmintic drug that have different chemical agent to prevent further drug resistance should be considered. The promotion of bull health management is highly recommended to protect humans from zoonotic diseases.

## Introduction

The raising of bulls for bullfighting is very popular in South Thailand. Fighting bulls are bred from indigenous cattle by searching for animals with distinctive properties. Bulls with the desired characteristics are raised on specialized ranches equipped with mosquito nets. The main feed includes all types of grass. The trainer walks the animals every morning at dawn for a distance of 5-10 km. After walking and exercising, the trainer takes the bull for showering and sunbathing to improve patience and strength. Although the raising of fighting bulls is more specialized than that of other types of cattle, in most cases, the owners lack the knowledge and understanding to look after and prevent several types of animal diseases. However, to keep the bulls competitive, the owner must pay attention to the bulls’ health. The owners, therefore, bring their bulls for initial physical check-ups at the animal hospital. However, local veterinarians may not have sufficient basic knowledge of the treatment and prophylaxis of infections recommended for the raising of fighting bulls.

In South Thailand, the number of bulls is approximately 10,000 [1]. Several health problems are observed in the animal hospital, including lameness, physical injury from fighting, infections, blood parasite infections, and internal parasitic infections. However, data on internal parasitic infections in fighting bulls in South Thailand remain limited. In addition, in dairy and beef cattle, there are only a few reports on gastrointestinal (GI) parasite infections, which is considered a major problem in livestock raising. In the Khon Kaen Province, a GI parasite infection prevalence of 53.34% has been reported in beef cattle [2]. The reported prevalence in cattle in slaughterhouses of Maha Sarakham Province was 93%, and these animals were infected mainly by helminths belonging to *Strongyles* spp., *Fasciola* spp., and *Moniezia* spp. [3]. In dairy cattle, the overall prevalence of GI parasitic infection in Thailand was reported to be 46.6%, with the highest infection rate in the south (98.4%); meanwhile, in the northern, central, and northeastern parts of the country, infection rates of 66.4%, 18.2%, and 16.7%, respectively, were observed. The GI parasites identified included rumen fluke, strongyles, *Trichuris* spp., *Moniezia* spp., *Eurytrema* spp., *Fasciola* spp., and *Eimeria* spp. [4]. The reported overall prevalence of enteric parasites in heifers and heifer calves in North Thailand was 54%; most of these parasites were trematodes (41%) and nematodes (26%) [5].

With regard to the prevalence of GI parasitic infections in cattle according to region, the highest prevalence has been found in the southern region of Thailand. The impact of GI parasite infection in cattle varies from mild to severe clinical symptoms. Mild or subclinical symptoms include losses in animal productivity such as reduced milk production and weight gain, altered carcass composition, and decreased conception rate. Moderate-to-severe symptoms include hair coat roughness, anemia, edema, and diarrhea. Even subclinical effects can be of major economic importance to the bull farmers.

Therefore, this study aimed to identify the prevalence of GI parasite infection in fighting bulls in South Thailand, thereby providing data that can inform future deworming programs.

## Materials and Methods

### Ethical approval

This study was conducted on 1501 animals at the animal hospital of Prince of Songkla University under Animal Ethics of Thailand (Approval no. U1-02915-2559).

### Sample collection and study period

Fecal samples were collected from 1501 fighting bulls aged 2 to 5 years and presented for physical health examination at the Prince of Songkla University Animal Hospital from November 2016 to October 2019. The IDs and breeding areas of the bulls were also recorded.

### Laboratory methods

This study used a standard protocol for the formalin ether concentration technique to detect parasite eggs [6]. Five grams of feces were dissolved in 15-20 mL of water. The dissolved feces were filtered into a tube using a mesh sieve and centrifuged at 2500 rpm for 5 min. The supernatant was discarded and 7 mL of 10% formalin solution was added; the tube was vortexed vigorously until the sediment was dissolved and then set aside for 5 min. Diethyl ether solution (3 mL) was added and the tube was vortexed well for 1 min followed by centrifugation at 2500 rpm for 5 min. The supernatant was discarded and 1-2 mL of 10% formalin solution was added to the remaining pellet. The pellet was suspended, and one drop was subsequently placed on a slide with a coverslip and examined with a light microscope at 100x and 400x magnification for screening of parasite eggs and protozoan cysts, respectively.

### Morphological identification and quantification of parasite eggs and protozoan cysts

Eggs of helminths and (oo)cysts of protozoa were identified by microscopy based on the morphological identification keys described by William [7].

### Statistical analysis

Data were analyzed as percentages using Microsoft Excel (Microsoft, Washington DC, USA) and further by descriptive analysis.

## Results

**Figure-1:**
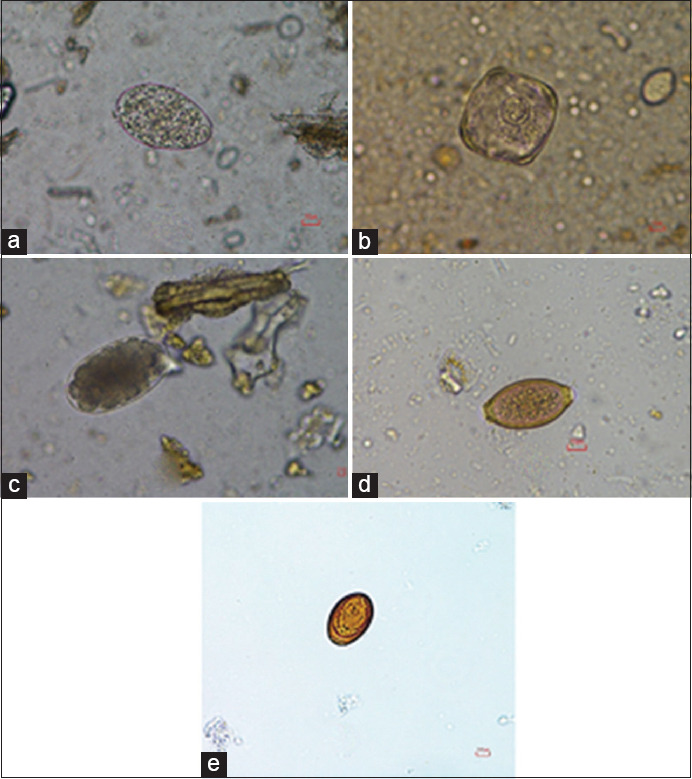
Helminth eggs present in fighting bull samples. (a) Rumen fluke; (b) *Moniezia* spp.; (c) *Strongyle* spp.; (d) *Trichuris* spp.; and (e) *Eurytrema* spp.

**Figure-2:**
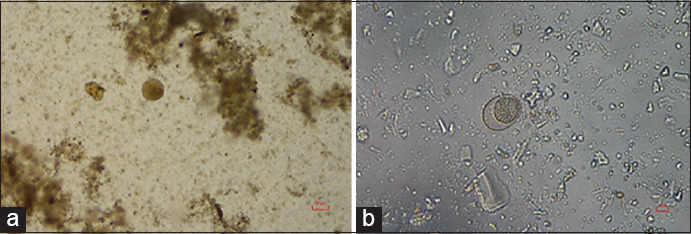
Oocysts present in fighting bull samples. (a) *Balantidium coli*; (b) *Coccidia* spp.

Out of the 1501 fecal samples, 1415 (94.27%) were positive for at least one enteric parasitic genus; coinfection with up to seven genera was seen. The most commonly observed GI parasite eggs were those of rumen fluke (97.17%), followed in frequency by strongyles (26.29%), *Eurytrema* spp. (2.83%), *Fasciola* spp. (2.47%), *Trichuris* spp. (0.35%), and *Moniezia* spp. (0.14%) (Table-1 and Figure-1). In addition to parasite eggs, protozoan cysts were also found, including those of *Balantidium coli* (6.64%) and *Eimeria* spp. (3.53%) (Table-1 and Figure-2). A total of 25 different types (33.99%) of parasitic coinfection were observed. The top five most common coinfections were those of rumen fluke with strongyles (20.85%), *B. coli* (4.66%), *Eimeria* spp. (1.55%), *Eurytrema* spp. (1.34%), and *Fasciola* spp. (1.06%) (Table-2).

**Table-1:** The gastrointestinal parasites found in 1415 positive samples of fighting bulls.

Type of parasite	Number of positives (%)	Public health concern
Nematode		
Strongyles	26.29 (372/1415)	Zoonotic parasite
*Trichuris* spp.	0.35 (5/1415)	Zoonotic parasite
Trematode		
Rumen fluke	97.17 (1375/1415)	None
*Eurytrema* spp.	2.83 (40/1415)	Zoonotic parasite
*Fasciola* spp.	2.47 (35/1415)	Zoonotic parasite
Cestode		
*Moniezia* spp.	0.14 (2/1415)	None
Protozoa		
*Balantidium coli*	6.64 (94/1415)	Zoonotic protozoa
*Coccidia* spp.	3.53 (50/1415)	Zoonotic protozoa

**Table-2:** Coinfection patterns of gastrointestinal parasite positive in fighting bulls (1415 samples).

Order	Pattern	Total no. of coinfections	Percentage
1	RF + ST	295	20.85 (295/1415)
2	RF + Bc	66	4.66 (66/1415)
3	RF + Ei	22	1.55 (22/1415)
4	RF + Eu	19	1.34 (19/11415)
5	RF + FS	15	1.06 (15/1415)
6	RF + ST+ Bc	14	0.99 (14/1415)
7	RF + ST+ Ei	10	0.71 (10/1415)
8	RF + ST+ Eu	12	0.85 (12/1415)
9	RF + ST+ FS	6	0.42 (6/1415)
10	RF + Bc+ Ei	4	0.28 (4/1415)
11	RF + ST+ Tri	1	0.07 (1/1415)
12	RF + ST+ Mo	1	0.07 (1/1415)
13	RF + Ei+ FS	1	0.07 (1/1415)
14	RF + Ei+ Eu	1	0.07 (1/1415)
15	RF + Bc+ FS	1	0.07 (1/1415)
16	RF + Bc+ Eu	1	0.07 (1/1415)
17	RF + ST+ Bc+ Eu	2	0.14 (2/1415)
18	RF + ST+ Bc+ FS	1	0.07 (1/1415)
19	RF + ST+ FS+ Tri	1	0.07 (1/1415)
20	RF + St+ Bc+ Cc	1	0.07 (1/1415)
21	RF + ST+ Eu+ Tri	1	0.07 (1/1415)
22	RF + ST+ Cc+ Mo	1	0.07 (1/1415)
23	RF + ST+ Cc+ FS	1	0.07 (1/1415)
24	ST + Ei	1	0.07 (1/1415)
25	ST + Bc	3	0.21 (3/1415)
		481	33.99

RF = Rumen fluke, ST = *Strongyle* spp., Bc = *Balantidium coli,* Ei = *Eimeria* spp., Eu = *Eurytrema* spp., FS = *Fasciola* spp., Tri *= Trichuris* spp., Mo = *Moniezia* spp.

## Discussion

As there are no data available on GI parasite infection in fighting bulls, this study aimed to identify the prevalence of GI parasite infections in such animals with the aim of supplying data that can be used to inform choices of anthelmintic drugs for bulls. Moreover, the data serve to identify the extent of the reservoir of zoonotic parasitic infections represented by fighting bulls. The data indicated a high prevalence of GI parasite infections in bulls (94.27%); however, the results differed from those in beef cattle in the Nan Province (61%) [8]. Our findings indicated that the most common parasite eggs in bulls were those of rumen fluke, which has a worldwide distribution and is considered an important parasite in ruminants [8,9]. This parasite does not cause clinical disease, but a large number of immature flukes in the rumens of young stock may result in severe enteritis and chronic diarrhea. In severe cases, it can cause fatalities in both cattle and sheep. There are a growing number of reports of animal morbidity and mortality associated with acute paramphistomosis (rumen fluke infection) in Europe [10]. Large numbers of rumen fluke eggs typically indicate that the bulls are fed contaminated fresh grass. Therefore, avoiding cutting the grass near water sources that may harbor intermediate host snails should be recommended to the bulls’ owners.

Coinfection with GI parasite mostly involved rumen fluke with parasites such as strongyles, *B. coli*, *Eimeria* spp., *Eurytrema* spp., and *Fasciola* spp., some of which are zoonotic. These results imply that parasite infections in bulls may be a reservoir of human GI parasitic infections. The prevalence of strongyle nematode infections (26.29%) in this study was higher than that observed in a previous study in cattle (10.76%) in Udon Thani, Thailand [11]; meanwhile, the overall prevalence of GI strongyle infection observed in Mindanao, Philippines, was 53% for cattle and 28% for buffaloes [12].

There were two species of trematodes identified in the bull samples, *Eurytrema* spp. (2.83%) and *Fasciola* spp. (2.47%). *Eurytrema* spp. are pancreatic flukes found in the pancreatic ducts and occasionally the bile ducts of sheep, pigs, and cattle [13-15]. Infections by these pathogens have been reported as potentially zoonotic [16]. *Fasciola* spp. are associated with diseases in cattle, sheep, and goats [17]. Although this study found a low prevalence of *Fasciola* infections, fasciolosis has been identified by the WHO as a reemerging neglected tropical disease associated with endemic and epidemic outbreaks of diseases in human populations.

The protozoa found in coinfections with rumen fluke in the bulls were *B. coli* and *Eimeria* spp. *B. coli* is a common opportunistic protozoan of man and animals and causes gastroenteritis, also known as balantidiasis, which mostly arises by ingesting infective cysts from food and water contaminated by feces from pigs or cattle [18]. Human infection is usually asymptomatic, but sometimes symptoms such as diarrhea and abdominal pain are observed [19]. There are reports of opportunistic infection by *B. coli* in HIV patients [20,21]. *Eimeria* spp. are parasitic protozoa that cause human coccidiosis, found mostly in tropical and subtropical regions. This affects people of all ages and has an important impact on public health. The infections may be symptomatic, depending on the population. Symptoms may include diarrhea, cramps, bloating, nausea, and vomiting and may be prolonged [22]. Disease caused by opportunistic *Eimeria* spp. is a common problem in HIV infection which the pooled prevalence of Isospora was 2.5% (788/105,922; 95% CI: 2.1-2.9%) [23].

Several risk factors affect the level of GI parasitic infection in cattle, such as the season, pasture area, age, breed, and parasitic drugs used as well as the presence of snails on the farmland [2,24,25]. The high rate of GI parasite infection in the southern region of Thailand may be due to high rainfall, which promotes the growth of the intermediate snail host on the pastures that are sources of bull feed. In addition, it was found that farmers often do not have their own grass plots and rather cut fresh grass near canals or other types of water bodies to feed their cattle. Therefore, methods of controlling GI parasites should be developed to fit individual bull production circumstances. Effective strategic deworming begins with understanding the life cycle and the potential disease burden of parasites. A successful deworming program, along with good overall herd management, will increase bull production and growth rate.

Some of the identified GI parasites have zoonotic potential. Therefore, the information should be distributed to the bulls’ owners and their family members so that they understand the dangers of those parasites. Good hygiene should be practiced by individuals who have close contact with the bulls to avoid infection by zoonotic parasites.

## Conclusion

This study is the first report of GI parasitic infections in fighting bulls in South Thailand. The high prevalence of GI infections reflects the potential risk of substantial economic losses in livestock production and determines the reservoir of zoonotic parasite infections in humans represented by these animals. Future studies should focus on the relationship between types and levels of parasitism with fighting endurance and animal production, and evaluate the parasitic dynamics throughout the year and the potential paths of spread to humans.

## Authors’ Contributions

RN planned and designed the experiment, data analysis, drafted, and revised the manuscript. DK collected the samples, conducted the experiment, and interpreted the results. RW collected the samples and conducted the experiment. SI conducted the experiment and interpreted the results. All the authors have read and approved the final manuscript.

## Competing Interests

The authors declare that they have no competing interests.

## Publisher’s Note

Veterinary World remains neutral with regard to jurisdictional claims in published institutional affiliation.
